# Modern Sociology

**Published:** 1900-10-27

**Authors:** 


### MODERN SOCIOLOGY.

### FACTORY LEGISLATION IN VICTORIA.

Our colonies are much more ready than ourselves to make legislative experiments, and the Colony of Victoria has this year passed a new Factories and Shops Act which is far in advance of anything that we have in Britain. It is too early yet to say how the Act will affect the condition of the workers, but it may be worth while to narrate the steps by which the legislature has been led to pass an Act which fixes a minimum wage for almost every trade, and makes the word "factory" cover even the smallest workshop. The first Victorian Factory Act was passed in 1874. It defined a factory as a place where not not less than 10 persons were employed. Women's work was limited to 8 hours a day; but the Chief Secretary had power to suspend that clause.

The weak points of this Bill were that it is in the smallest workshops that the conditions of labour are worst, and that, even if the 8 hours' limit for women was carried out, there was no limitation of "home work"—that which is the most ill-paid, and in which the hours worked are the longest. A strike of workgirls in the clothing trade brought these facts before the public, and a tailoresses' trade union was formed which laid down rules as to hours and wages. A Commission was also appointed to inquire into the conditions of labour in 40 trades. This Commission brought out its report in 1874. The report showed that the average hours of labour for males ran from 8½ to 13¼, though in some cases 15 and even 18 hours had been worked per day. For women the average was from 8 to 12 hours, the maximum being 16. But the Commission had not been able to investigate the conditions in the smallest workshops, where "sweating" is always at its worst, nor had they been able to make a complete inquiry into the conditions of home work.

The minimum wage fixed by the new tailoresses' union was nominally adopted by the employers, but they managed to evade its clause. By giving out home work and by means of the apprentice system the employers were able, if they so willed, to nullify in great measure the effect of the Act. The Commission recommended that no work should be given to be done at home, "except under pressure"—an exception that might undo all the good of the regulation. The existing Act fixed no limit to the number of apprentices that might be taken. It was fixed indeed by the "log" of the tailoresses that apprenticeship should be for two years, with wages rising from 2s. 6d. to 12s. a week; but when the period of apprenticeship was completed the girls were generally placed on piecework at low wages, and when they had become proficient enough to demand the standard rate of wages for skilled workers they were dismissed, and only taken on again if they would consent to come as "improvers" at low wages. The failure of the union to enforce their wage-rate shows how difficult it is to fight low wages by direct action.

After the report of the Commission appeared, a new Factory Act was passed in 1885, but it showed little advance on previous legislation. The chief improvement consisted in reducing the number of persons constituting a factory from ten to six; but this only if all the persons working in the place did not belong to the same family. At this point factory legislation remained for ten years.

In 1895 a new Bill was passed by the Lower House of Parliament, but was rejected by the Upper. It had embodied the principle of the minimum wage, appointed trade boards to regulate prices, and regulated shop hours and holidays. It contained also a provision for the inspection of indoor work, and it was on this that it was wrecked in the Upper Chamber. But before Parliament reassembled it became evident that the people of Victoria had set their heart on the Bill. The Metropolitan Board of Works had already adopted a minimum rate of wages of 6s. a day for men between the ages of twenty and fifty-five. In 1896 the Bill was introduced again, and this time it became law. One of its provisions has a certain interest for us now, since we also are threatened with an inroad of Chinese labour. It ran that "*four* persons other than Chinese, and *one* Chinese should constitute a factory." This was evidently to prevent these aliens from working under the insanitary conditions which make their presence a public danger, as well as an unfair handicap on native workers. The Bill also provided that a record of outside work should be kept, with the names and addresses of the workers. Powers were given to appoint boards to fix the minimum rate of wages in various trades, half the members of the board to be elected by employers and half by employed.

About this time an "anti-Sweating League" was formed, which devoted itself to investigating the degree to which the regulations of the Factories Act were evaded. The League found that, especially in those trades where there was no board to fix wages, these fell far below the minimum wage in other trades. The secretary of the League was able to produce sworn affirmations from pastrycooks, one of whom had been working 106 hours a week for 30s., and another 90 hours a week for £1. Men in the butchering trade were working from 70 to 90 hours a week for wages varying from 12s. to 17s. 6d. a week. The "white workers" or underclothing makers had no board, and there it was shown that the out-workers in it were paid from 25 to 50 per cent. less than those in factories. The dressmakers had no board, and there it appeared that 3,813 out of a total of 4,554 workers were earning only 8s. 7d. a week. Yet the tailoresses and the women in other trades protected by boards were earning a minimum of 16s. Apprentices in the dressmaking and millinery trades were also grossly cheated. Miss Cuthbertson, Inspector of Factories, found out that in many cases the 2s. 6d. a week which the apprentice was legally bound to receive was not paid at all, but kept back, "for value received," whatever that might be. In others it was paid, but had to be handed back at once; in yet others the girls were allowed to keep it from Saturday to Monday, when they had to return it. In one case a girl had paid a "premium" of £5 to enter the trade. She was paid 2s. 6d. a week for six months and then dismissed, being thus cheated out of 35s., besides having given six months' work for nothing.

To meet these evils an Act was passed this year extending the minimum wage principle to almost all trades, authorising the Governor in Council to appoint boards to fix the wage, and making regulations for the inspection of all places where work is done for trades under these boards, whether they be factories or not. "Contracting out" of the fixed wage is forbidden, as is also the "truck" system and the paying of a premium by apprentices in the clothing trades. The hours of work are limited to fifty-two per week for males as well as females. The Act is passed for two years only. At the end of that time it will be interesting to find out if it has so far diminished the evils it is meant to attack as to justify its being made permanent.

